# A New Measure for Neural Compensation Is Positively Correlated With Working Memory and Gait Speed

**DOI:** 10.3389/fnagi.2018.00071

**Published:** 2018-03-19

**Authors:** Lanxin Ji, Godfrey D. Pearlson, Keith A. Hawkins, David C. Steffens, Hua Guo, Lihong Wang

**Affiliations:** ^1^Center for Biomedical Imaging Research, Department of Biomedical Engineering, Tsinghua University, Beijing, China; ^2^Olin Neuropsychiatry Research Center, Institute of Living, Hartford Hospital, Hartford, CT, United States; ^3^Department of Psychiatry, Yale School of Medicine, Yale University, New Haven, CT, United States; ^4^Department of Neuroscience, Yale School of Medicine, Yale University, New Haven, CT, United States; ^5^Department of Psychiatry, University of Connecticut Health Center, Farmington, CT, United States

**Keywords:** neural compensation, cognitive reserve, aging, ICA, gait speed, fMRI methods

## Abstract

Neuroimaging studies suggest that older adults may compensate for declines in brain function and cognition through reorganization of neural resources. A limitation of prior research is reliance on between-group comparisons of neural activation (e.g., younger vs. older), which cannot be used to assess compensatory ability quantitatively. It is also unclear about the relationship between compensatory ability with cognitive function or how other factors such as physical exercise modulates compensatory ability. Here, we proposed a data-driven method to semi-quantitatively measure neural compensation under a challenging cognitive task, and we then explored connections between neural compensation to cognitive engagement and cognitive reserve (CR). Functional and structural magnetic resonance imaging scans were acquired for 26 healthy older adults during a face-name memory task. Spatial independent component analysis (ICA) identified visual, attentional and left executive as core networks. Results show that the smaller the volumes of the gray matter (GM) structures within core networks, the more networks were needed to conduct the task (*r* = −0.408, *p* = 0.035). Therefore, the number of task-activated networks controlling for the GM volume within core networks was defined as a measure of neural compensatory ability. We found that compensatory ability correlated with working memory performance (*r* = 0.528, *p* = 0.035). Among subjects with good memory task performance, those with higher CR used fewer networks than subjects with lower CR. Among poor-performance subjects, those using more networks had higher CR. Our results indicated that using a high cognitive-demanding task to measure the number of activated neural networks could be a useful and sensitive measure of neural compensation in older adults.

## Introduction

With the global increase in the aging population, there is an urgency to better understand neural mechanisms of age-related cognitive decline and resilience in order to promote healthy brain aging (Christensen et al., 2009). While brain regional volume loss typically occurs with aging, brain reorganization in older adults may compensate for neural deterioration (Gutchess, 2014; Morcom and Johnson, 2015). Functional neuroimaging studies have reported that, compared with young adults, neural activity increases in older adults in a variety of aging-vulnerable brain regions including the prefrontal cortex, posterior parietal cortex and right parahippocampal gyrus/lingual gyrus (Rajah and D’Esposito, 2005; Greenwood, 2007; Park and Reuter-Lorenz, 2009; Steffener et al., 2009; Lighthall et al., 2014). To accommodate these changes in activity, the brain may be able to reorganize its functioning to counteract neural decline and to maintain performance (Chanraud and Sullivan, 2014). In many studies, this over-activation is accompanied by better performance, raising the possibility that the additional activity serves a beneficial, compensatory function without which performance decrements would result. However, the processes underlying neural compensation and how it benefits cognitive performance remain unclear (Morcom and Johnson, 2015).

A notable construct related to neural compensation is cognitive reserve (CR). The concept of CR suggests that innate intelligence or aspects of life experience may supply reserve, in the form of a set of skills or repertoires that allows some people to cope with progressing Alzheimer’s disease pathology better than others (Scarmeas and Stern, 2003). From the perspective of neuroimaging, the following definition of CR is proposed: the ability to optimize or maximize performance through differential recruitment of brain network, which perhaps reflect the use of alternative cognitive strategies. CR may be based on either more efficient utilization of brain networks or of enhanced ability to recruit alternate/compensatory brain networks (Stern, 2002). Better understanding of neural compensation would support the concept of CR. Typically, CR is measured indirectly by experiences across the lifespan, including educational and occupational attainment, leisure activities and IQ (Stern, 2012). However, these CR proxies are themselves correlated with each other and they cannot show changes with time. Reed et al. (2010) recently proposed to quantify CR using a residual memory score after regressing out memory components corresponded to demographic and brain structural variables. Since CR has been shown to predict development of Alzheimer disease, it is important to examine whether neural compensatory ability contributes to CR. In the present study, we examined the relationship between the number of activated neural networks and CR defined by Reed et al. (2010). We propose that the relationship between CR and compensatory ability measured by the number of activated neural networks may differ depending on challenging task performance. When task performance is poor (or there exists clinical symptoms of cognitive impairment), CR should be reduced compared with a condition of good task performance with preserved cognitive function. Among subjects with poor task performance, those who have activated more networks during performing the task should have higher CR than those with fewer networks due to limited brain resources. On the other hand, among subjects with good task performance, those who have fewer networks (need less effort to complete the task) should have higher CR than those activated more networks.

Few studies have investigated factors influencing compensatory function beyond educational level and occupational attainment. Physical activity level, including gait speed is important to life quality of older adults. Physical exercise improves executive function (Colcombe and Kramer, 2003; Lautenschlager et al., 2008; Ji et al., 2017), and gait ability has been shown to be associated with global and executive cognitive function (Atkinson et al., 2007), as well as the activation level of executive network in older adults (Jor’dan et al., 2017). These findings support studying the relationship between gait speed and neural compensatory capability.

Therefore, we hypothesized that, if the number of activated neural networks can reflect neural compensation, then: (1) greater damage to core task-relate neural networks should evoke greater number of neural networks as a compensatory mechanism; (2) those individuals who have activated more networks should have better cognition than those who lost capacity to activate additional networks; and (3) factors such as physical activity and gait speed might influence brains’ compensatory function.

## Materials and Methods

### Subjects

Participants included 26 cognitively normal adults (age: 73.23 ± 6.68, male/female = 1) and older, who were either retired professors from Tsinghua University or their spouses, the latter having education levels less than 16 years. Exclusion criteria for the study were: (1) magnetic resonance imaging (MRI) contraindications and claustrophobia; (2) severe or unstable medical disorders, conditions, or drugs that may cause any condition that in the investigators’ opinion might make the patients unsuitable for participating in the study (e.g., clinically significant cirrhosis, or heart disease); (3) any known current or past diagnosis of psychiatric disorders; (4) active suicidality or current suicidal risk; (5) significant handicaps that would interfere with neuropsychological testing or the inability to follow study procedures; and (6) any other factor that in the investigators judgment may affect patient’s safety or compliance.

We used a score of 26 and below on the Mini-Mental State Examination (MMSE: 28.8 ± 0.8) to exclude those with possible mild cognitive impairment (MCI). The study protocol was approved by Institutional Review Board of Tsinghua University School of Medicine. All participants were well informed about the study and gave written informed consent.

### Assessments of Cognitive Function, Mood and Physical Activity

In addition to the MMSE, a neuropsychological test battery was used to assess cognitive function for all subjects. The battery included the Chinese Rey Auditory Verbal Learning Test (RAVLT; Guo et al., 2009), Chinese version of Logical memory subtest of the Wechsler Memory scale (LMT; Guo et al., 2009), WAIS-III Digit-Symbol Substitution Modality Test (DSST), WAIS-III Digit span (Wechsler, 1997), Trail Making Test (Trails A and Trails B; Reitan, 1958), and Benton visual retention test (BVRT; Benton, 1974). Subjects’ performance scores were standardized based on normative data (Gong, 1992). Subdomains were assessed using RAVLT, LMT, BVRT and delayed recall scores for memory; Trails B for executive function; DSST and Trails A for information speed; and Digit span for working memory.

Mood state was evaluated using the Positive and Negative Affect Schedule (PANAS; Watson and Clark, 1999), the Geriatric Depression Scale (GDS; Yesavage and Sheikh, 1986), and the Profile of Mood State (POMS; Grove and Prapavessis, 1992). Tension, depression, anger, vigor, fatigue and confusion sub-scores of POMS were calculated.

The International Physical Activity Questionnaire (IPAQ^1^) was used to evaluate subjects’ basic physical activity level. A six-min walking test (6MWT) was employed to examine gait speed (Enright, 2003) as the level of physical activity. Twenty-two subjects completed the 6MWT.

### Imaging Acquisition

MRI scans were conducted at Tsinghua University on a Philips 3 Tesla (T) TX Achieva scanner (Philips Healthcare, Best, Netherlands) for each participant. During each neuroimaging session, the following image acquisition protocols were used. First, sagittal T1-weighted spin-echo images were collected to identify landmarks for reference. Second, a T1-weighted sequence provided high-resolution anatomical images with 180 slices, slice thickness = 0.9 mm, acquisition matrix = 256 × 256 × 180. Third, task-related fMRI was acquired using EPI sequence with the following parameters: TR = 2000 ms, TE = 30 ms, flip angle = 80 degree, acquisition matrix = 64 × 64 × 34 slices, voxel size = 3.5 × 3.5, slice thickness = 3.5 mm with a gap of 0.5 mm. A total 188 volumes were acquired.

**Figure 1:**
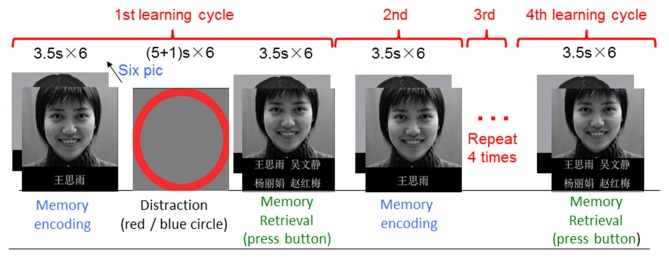
Illustration of the fMRI task. The task was modified from Zeineh’s article (Zeineh et al., 2003). The pictures were taken from the Chinese Facial Affective Picture System (Gong et al., 2011).

The memory task was a block-event mixed design (Figure 1), which included memory encoding, memory retrieval and distraction blocks. In a memory-encoding block, there were six face-name pairs presented sequentially. Each face was associated with a name beneath it. Subjects were asked to remember the faces and associated names during a memory-encoding block, and to choose the correct name (out of four name choices) associated with a face during a memory-retrieval block. The same encoding and retrieval block pairs were repeated four times to examine subjects learning ability. The task was modified from Zeineh’s article (Zeineh et al., 2003). The pictures were taken from the Chinese Facial Affective Picture System (Gong et al., 2011). Typically, subjects should remember the names associated with the faces better in the later retrieval blocks compared with the first retrieval block since the same face-name pairs were presented four times repeatedly. In-between an encoding and a retrieval block, there was a Go/No-Go distraction block. During a distraction block, there were “+” signs following with a red circle or blue circle randomly. There were six “+” “circle” pairs in one distraction block (three circles in red, and three circles in blue). Subjects were asked to press a button as quickly as possible when they saw a red circle, and withhold the button when a blue circle appeared. Each face-name pair lasted for 3.5 s. Each block lasted for 21 s. During a distraction block, each “+” sign lasted for 5 s, and each circle lasted for 1 s. Therefore, one task run lasted for 6.2 min. The task not only allowed us to examine memory encoding and memory retrieval, it also allowed us to assess target executive function and inhibition. There were three task runs in total with pictures in different facial expressions of positive, neutral and negative.

### MRI Data Processing

#### MRI Structural Data Preprocessing and Analysis

The brain parcellation and segmentation were conducted using the standard recon-all script of Freesurfer package^2^ (Fischl, 2012) with default settings for all procedures. Volumes of 97 subcortical regions and 68 cortical areas from Freesurfer recommended template were included as region of interests. Out of these ROIs, we focused on the ratio of hippocampus volume/temporal horn volume, as a structural index related to memory for CR calculation. The whole-brain gray matter (GM) volumes, total intracranial volumes and white matter hyper-intensity (WMH) volumes were also measured for CR calculation.

#### Task-Related fMRI Data Preprocessing and Quantification of Compensatory Function

The fMRI data was preprocessed using CONN software 17.a (Whitfield-Gabrieli and Nieto-Castanon, 2012) based on SPM8 Toolbox^3^. The pre-processing steps included realignment, slice-timing correction, registration of functional images to structural images, normalization to standard space, and smoothing with a Gaussian kernel of 8 mm FWHM. Then we used GIFT toolbox^4^ to conduct independent component analysis (ICA; Salimi-Khorshidi et al., 2014) on the pre-processed data. fMRI BOLD signals across all three runs (75 fMRI scans in total) were decomposed into 45 components based on the estimation on data quality using MDL criteria (Li et al., 2007). Among them, 15 components were identified as signals according to the principles described in Salimi-Khorshidi et al. (2014).

#### Number of Activated Networks During the Memory Task

In order to examine how many neural networks were activated during performing the task, we examined the relationship between time-courses of each component identified as signals and a predicted hemodynamic response (with double-gamma HRF) of our task design. The components that were significantly correlated (*p* < 0.001) with the hemodynamic response of the task design were identified and these components were counted as the number of activated neural networks for each subject. Given that the significant threshold (*p* < 0.001) was arbitrarily selecting, we further examined how threshold used to define active networks affect our results by repeated the analysis procedures using a wide range of thresholds including *p* = 0.0001 (*r* = 0.2811), *p* = 0.0005 (*r* = 0.2520), *p* = 0.001 (*r* = 0.2383), *p* = 0.005 (*r* = 0.2040) and *p* = 0.01 (*r* = 0.1875).

The probability of being activated for each component was calculated using the ratio of occurrence of “activated networks” divided by 75 (the number of total scans). We defined the top three commonly activated components as core networks related to the memory task. Pearson’s correlation analyses were conducted between the compensatory capacity (defined later in results) to cognitive function, mood state and physical activity level. The significant level for correlations to cognitive function was set as *p* < 0.0125(0.05/4 cognitive domains) to correct for multiple comparisons.

### Cognitive Reserve Measurement

We measured CR according to Reed’s methods, whose details are available in Reed et al. (2010). A latent variable model was created in Mplus application (Muthén and Muthén, 2007). In brief, memory function was decomposed into components corresponding to demographic (MemD), MRI variables (MemB) and a residue (MemR). Memory function was the average of RAVLT score (average of the first five trials), LMT, and BVRT. Observed demographic variables included in the model were years of education and indicators for gender. Observed MRI variables included total GM volumes, hippocampal volumes and total WMH volume. All MRI variables were standardized before included into the model. Total GM volume was regressed out with total intracranial volume. Similarly, the hippocampus volume was adjusted for the adjacent temporal horn volume. White matter hyperintensity was also modeled through a latent variable, but was not adjusted by intracranial volume.

### Relationship Between Compensatory Function With CR

To further explore the relationship between the number of networks and CR, subjects were split into four subgroups based on the median of correct rate of memory task performance (median for accuracy = 0.31) and the number of activated networks (median for number of networks = 7.4): (1) poor performance, fewer networks; (2) poor performance, more networks; (3) good performance, fewer networks; and (4) good performance, more networks. We compared CR across groups with the average as well as median/quarter value.

## Results

### Cognitive and Behavioral Results

Table 1 shows the demographic profile, memory task performance, mood state and results of neuropsychological tests. As shown in the table, the averaged correction rate in recognized face-name association was 33%. The low memory accuracy was possible due to the distraction of the Go/No-Go task between a memory encoding and a memory retrial block. The results indicate that this task was quite difficult and challenging for older adults.

**Table 1:** The demographic profile of participants and cognitive function at baseline (*n* = 26).

	Group mean (STD)
**Age (years)**	73.23 (6.68)
**Sex (M/F)**	13/13
**Dominant hand (R/L)**	25/1
**Education (years)**	15.19 (2.73)
**Mood state (*n* = 24)**	
PANAS—positive	31.42 (6.93)
PANAS—negative	15.42 (4.62)
PMOS—tension	3.96 (3.32)
PMOS—depression	2.71 (3.21)
PMOS—anger	3.17 (4.12)
PMOS—vigor	13.12 (4.52)
PMOS—fatigue	4.5 (3.65)
PMOS—confusion	4.59 (2.69)
PMOS—self-esteem	4.58 (2.69)
GDS	5.46 (5.47)
**Cardiovascular function**	
6MWT (m) (*n* = 22)	405.04 (88.67)
IPAQ (MET-minutes/week)	4530.96 (2146.34)
**Neuropsychological test**	
Memory function	7.97 (1.29)
LMT (t)	8.58 (1.90)
Benton Visual memory Test (t)	6.88 (1.51)
AVLT (t)	8.44 (1.58)
Working memory function	13.65 (2.53)
WAIS-III Digit Span (t)	13.65 (2.53)
Information speed (*n* = 23)	27.11 (6.11)
DSST (t)	14.78 (2.80)
Trails A Making Test (t) (*n* = 23)	39.17 (10.13)
Executive function	38.95 (10.74)
Trails B Making Test (t) (*n* = 23)	38.95 (10.74)
**fMRI task performance**	
Accuracy rate in memory retrieval	0.33 (0.15)
Accuracy in Go/No Go task	0.83 (0.19)
Reaction time in Go stimuli (seconds)	0.60 (0.28)

*6MWT, Six-min walking test; IPAQ, International Physical Activity Questionnaire: scored by total energy requirements in metabolic equivalent (MET) to kilocalories for a 60 kilogram person in rest. PANAS, Positive and Negative Affect Schedule; PMOS, Profile of Mood States; GDS, Geriatric Depression Scale. LMT, Logical memory subtest of the Wechsler Memory scale. AVLT, Recall from Rey Auditory Verbal Learning Test; DSST, WAIS-III Digit-Symbol Substitution Modality Test. Accuracy in Go/No Go task: (True Positive + True Negative)/Total items*.

### Correlation Between the Numbers of Activated Networks and Brain Volumes of the Core Networks

**Figure 2:**
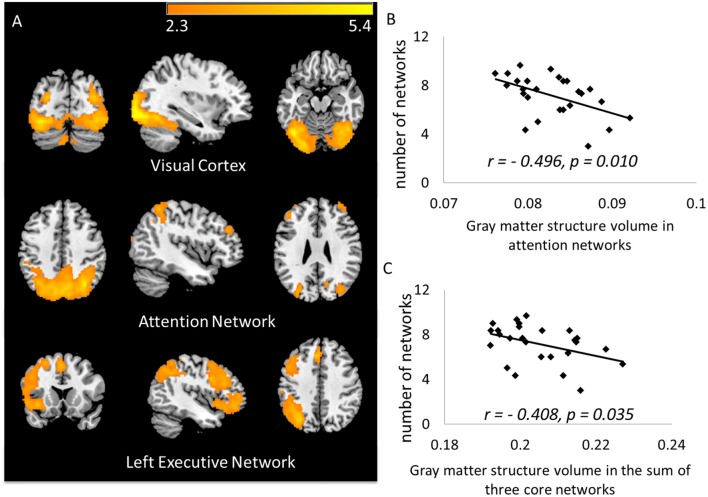
**(A)** The top three core networks derived from independent component analysis (ICA) that commonly activated in a majority of subjects. ICA maps were converted to *z* statistic images, thresholded at *z* > 2.3; **(B)** number of activated networks showed negative correlation to gray matter (GM) volume in the attention network (ATN); **(C)** number of activated networks showed negative correlation to GM volume in the total three core networks.

Across all 15 components identified as signals, the most commonly activated three networks were the visual network, part of the attention network, and the left executive network, with chances of being activated by 97.3%, 85.3% and 77.3% of participants (Figure 2A). Specifically, the following areas were identified in the three networks:

1. Visual network: bilateral visual cortex, bilateral fusiform gyrus;
2. Attention network: bilateral precuneus, bilateral superior parietal area, bilateral inferior parietal area and bilateral rostral middle frontal area;
3. Left executive network: left insula, pars opercularis of inferior frontal cortex, pars orbitalis of interior frontal cortex, caudal middle frontal area, rostral middle frontal area, superior frontal area, lateral orbital frontal area, superior temporal area and inferior parietal area.

Volumes of the discrete brain structures covered by each network were normalized by total intracranial volume, and then summed up as the volume of each network. As we predicted, the smaller volume of the three core networks, the more additional networks were activated during performing the task (*r* = −0.408, *p* = 0.035; Figure 2C). Separately, the number of activated networks are correlated with the attention network (Pearson *r* = −0.496; *p* = 0.010, Figure 2B), with the left executive network (*r* = −0.291; *p* = 0.149), and with the visual network (*r* = −0.171; *p* = 0.401). To avoid the influence of the atrophy in core networks, in subsequent analyses, we used the number of activated networks regressing out the volume of the three core networks as a measure for neural compensatory capacity.

In our face-name memory retrieval task, there were four name options for each face. We infer that those subjects who have a correct rate less than 25% might have completely failed in remembering the items, and just pressed the buttons by chance. Therefore, we excluded subjects whose correct rate was lower than 25% (*n* = 16 subjects remained) to recheck the results. As a result, the volume in the attention network (*r* = −0.559, *p* = 0.025) was still significantly correlated with the number of networks after excluding subjects with poor performance.

### Correlation Between Compensatory Function to Cognition and Fitness

Residue left in the number of networks after regressing out of volumes of the three core networks was used to quantify the compensatory capacity. To verify the benefit of compensatory network to cognition, and the influence of physical fitness, Pearson correlations were applied between the compensatory capacity to cognitive tests as well as to physical activity measures.

**Figure 3:**
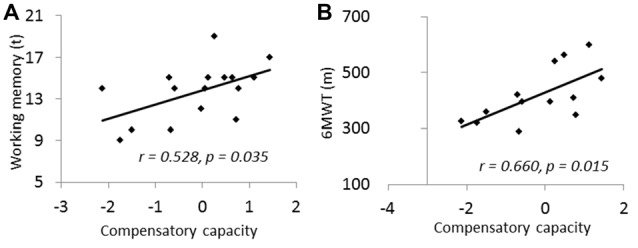
**(A)** Compensatory capacity showed positive correlation to working memory. **(B)** Compensatory capacity showed positive correlation to 6-min walking test (6MWT). Compensatory capacity was measured by number of activated networks controlled for the GM volumes in core networks.

No significant correlation was found between the compensatory capacity and cognitive function across all subjects. However, when only including those subjects who have accuracy rate above chance (more than 25%) during the task (*n* = 16), the compensatory capacity measured by proposed method was marginally correlated to working memory function (*r* = 0.528, *p* = 0.035; Figure 3A). Interestingly, the compensatory capacity showed a significant correlation to 6MWT (*n* = 13, *r* = 0.66, *p* = 0.015; Figure 3B). 6MWT was also correlated with working memory function across subjects with behavior performance above chance (*n* = 13, *r* = 0.68, *p* = 0.010) and across all subjects (*n* = 22, *r* = 0.48, *p* = 0.021).

### Role of Compensatory Capacity and CR in Predicting Behavior Performance

**Figure 4:**
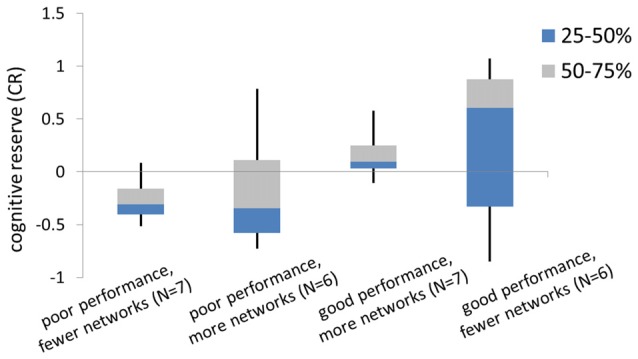
The box diagram of cognitive reserve (CR) in subgroups. Subgroups were split by the median number of the memory performance (recall accuracy) during the fMRI task, and the median number of the compensatory capacity.

First, there wasn’t a significant correlation between CR and compensatory capacity. We hypothesize that the relationship between CR and compensatory capacity differs depending on task performance. Indeed, when subjects were grouped by task performance and compensatory capacity, we found opposite relationship trends between CR to compensatory ability among good performance subjects vs. poor performance subjects. That is, “good performance, fewer networks” group showed higher CR than “good performance, more networks” group. While “poor performance, fewer networks” group showed lower CR than “poor performance, more networks” group. CR across groups with the average as well as median/quarter values is shown in Figure 4.

### Comparison of the Results Using Different Task Thresholds

To examine how threshold of significance might impact our results, we conducted the analyses following the same procedures using a range of thresholds in defining task-related networks, including *p* = 0.0001 (*r* = 0.2811), *p* = 0.0005 (*r* = 0.2520), *p* = 0.001 (*r* = 0.2383), *p* = 0.005 (*r* = 0.2040) and *p* = 0.01 (*r* = 0.1875). We found that the numbers of task-related networks survived at these thresholds were highly correlated to each other with *r* values from 0.878 to 0.978.

**Figure 5:**
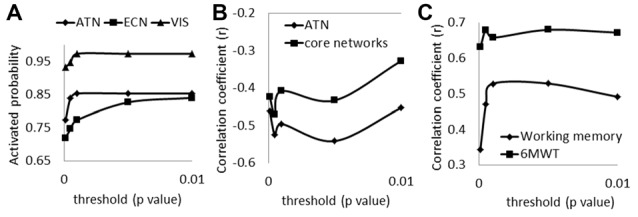
**(A)** The possibilities of being activated from all scans for the three core networks under different thresholds (ATN, attention network; ECN, left executive network; VIS, visual network); **(B)** correlations of the number of activated networks with the GM volumes in ATN and with the GM volumes in the total three core networks under different thresholds; **(C)** correlations of compensatory capacity with working memory function and with 6MWT under different thresholds (6MWT, 6-min walking test).

Under each of the thresholds we tested, the three most commonly activated networks remained the visual network, attention network and left executive network. Probabilities of being activated from all scans for the three core networks are shown in Figure 5A. Both the attention network and the visual network tended to be stable from a threshold at 0.001. Figure 5B shows the relationship between the brain volume of the core networks and the numbers of task-related networks with different thresholds. We found that there was a general compensatory mechanism for the atrophy of core networks with thresholds stricter than *p* = 0.005. Since the activated probability were not stable when the thresholds are stricter than *p* = 0.001, we recommend the threshold at *p* = 0.001.

We also tested the correlations between compensatory capacity to working memory score and to 6MWT (Figure 5C) under different thresholds. Results show that the significant correlations of the compensatory function to working memory and 6WMT maintained with *p* = 0.001 or lesser stringent thresholds.

## Discussion

In the present study, using an ICA approach, we found that among older cognitively intact adults performing a cognitively challenging task, there was a significant increase in the number of active neural networks when the task-related core networks were smaller in volume. We proposed the number of activated neural networks controlling for the volume of the core networks as a measure of neural compensatory capacity. Compensatory capacity was correlated with working memory, as well as the physical activity level measured by the 6-min walking test (6MWT). In addition, the relationship between the compensatory capacity and CR measured by CR was reversed between good and poor task performance. These findings suggest that the semi-quantitative measure of the compensatory capacity is valuable in the evaluation of individual variances of cognitive function.

Neural compensation refers to neural activation of additional brain regions or networks (and thus cognitive strategies) that typically are not activated. These additional networks typically are not engaged in performing a task until the task demands exceed the working capacity limit of task-related core networks (Steffener and Stern, 2012) or when the task-related neurons/networks were damaged. This explains why there was a significant correlation between lower volume of task-related core networks and greater number of activated neural networks. Similar to what is hypothesized by Chanraud and Sullivan (Chanraud et al., 2013), one can only detect a drop in compensation ability when the cognitive task is highly demanding. Therefore, a key to be able to measuring the neural compensatory capacity is to employ a highly cognitive demanding task. In our study, the mean task accuracy rate was 0.33, suggesting our memory task was very challenging to our study sample.

Previously identified patterns of aging-related compensatory activity were all based on comparisons between older and younger adults. One well-known pattern involves additional neural recruitment in bilateral prefrontal cortex in older adults with high performance in tasks that typically only activate one hemisphere in younger adults. This pattern is known as Hemispheric Asymmetry Reduction in Older Adults or HAROLD (Cabeza, 2002). In another pattern, the age-related increased activation (over-recruitment) in a set of regions including bilateral middle/superior frontal gyri, anterior medial frontal gyrus, precuneus and left inferior parietal lobe is coupled with age-related decreased activation (under-recruitment) in occipital and fusiform cortex during memory encoding process, which is known as Posterior-Anterior Shift with Aging or (PASA; Davis et al., 2007; Maillet and Rajah, 2014). Some studies have further revealed associations between greater activity and better task performance during fMRI scans (Dennis et al., 2007; Brassen et al., 2009) as well as higher cognitive functions (for review see Grady, 2008), which confirmed the beneficial effect of neural compensatory activity. While these patterns are helpful in understanding how neural recruitment are reorganized in older adults when engaging a cognitive task, they cannot be utilized to quantitatively assess compensatory ability. Particularly in patients with mental disorders who have deficits in some brain regions, such as in chronic alcoholism (Pfefferbaum et al., 2001), alternative networks/information processing strategy might be used, which explains why neural compensation patterns measured by activity differences within localized regions between age groups have raised controversies across reports. Therefore, we not only need to measure neural compensatory ability quantitatively, but also should assess neural compensation in system-wise and should take structural deficits into account. Our current study is the first to measure neural compensation using a data-driven method at the functional network level. This measure can be used to evaluate neural compensatory capacity for each individual subjects, which is useful for longitudinally tracking dynamic changes of the capacity over time. The compensatory capacity measured by our study method was correlated with task-related core structural atrophy and cognitive performance. In addition, it also showed interesting interactions with an existing quantitative measure of CR, which validated the measure as a proxy of CR.

However, our semi-quantitative measure of compensatory capacity has to be tied with task performance because poor task performance is clearly a sign of neural compensation failure. According to the Compensation-Related Utilization of Neural Circuit Hypothesis (CRUNCH model; Reuter-Lorenz and Cappell, 2008), older adults are likely to show over-activation as task demands increase. As working load increases to an extend that recruitment of neural resources have reached the limit of CR in some older adults, under-activation and poor performance would occur (Cappell et al., 2010; Reuter-Lorenz and Park, 2010). Therefore, dividing subjects based on good and poor task performance in our study is in consistent with the theory of Chanraud and Sullivan (2014) who have defined brain functioning changes in two types: (1) changes in brain function may be adaptive and enable successful compensation (successful compensation); or (2) change in brain function may be poorly preserved, resulting in unsuccessful compensation (attempted compensation). CR results based on four subgroups provided a good example of the CRUNCH model. That is, although “poor performance—more network” group could recruit as many neural networks as good performers, their task performance was still poor, indicating that the compensation was attempted but may not enough to support normal performance due to low CR. Conversely, subjects with good performance-more networks may have atrophy in some brain regions, yet their relatively higher reserve enabled them to achieve successful compensation. Whereas subjects without brain atrophy need less compensatory effort, and therefore they had good performance and fewer activated networks. Although small sample size in each group limited our conclusions from our current study, we believe the trends we found confirmed our theory on the relationship between CR and neural compensation capacity of brain aging. Of note, we used the median split to define good-performance and poor-performance groups due to the small sample size. Given that the median number would vary greatly among different study samples, using median split to define good and poor performance groups would reduce the feasibility for study validation. Future studies in a large study sample are needed to obtain population-based norm scores so that to define good and poor performance groups more accurately.

Another critical issue is the task used in evoking compensatory activation. Depending on tasks, the core networks would be different. Previous studies defined “primary networks” (or “core networks”) based on activation patterns in the younger group (Cabeza, 2002; Davis et al., 2007). Nyberg et al. (2010) demonstrated limitations of this approach by comparing 6-year longitudinal estimates to cross-sectional estimates. The cross-sectional study observed age-related frontal over-recruitment, whereas the longitudinal analysis revealed frontal under-recruitment with advancing age. Their finding indicated that results from cross-sectional studies could be influenced/biased by the study samples. To avoid sample bias, in our study, we distinguished core networks from additional/compensatory networks based on the frequency being activated by all study participants. In addition, our task not only allowed us to examine memory encoding and memory retrieval, it also allowed us to assess working memory, target executive function, and inhibition. It is quite reasonable that the visual network, attention network and left executive network were found as “core” fundamental networks for all subjects to complete a visual related task. We emphasize that beyond requiring a cognitively challenging task for measuring neural compensatory capacity, a task that covers all cognitive processing domains is also vital to detect one’s neural compensatory capacity globally. However, we do acknowledge that task designs in a specific cognitive domain are also important if there is a need to evaluate neural compensatory capacity in a specific cognitive domain.

While we examined compensatory capacity using a cognitively challenging task, it is also interesting for further studies to examine the neural compensatory mechanism during resting state. Frantzidis et al. (2014) have examined compensatory connectivity in MCI patients in terms of small-world network properties during resting state. It would also be intriguing to examine the link between CR and neural compensation in rest brains. As discussed above, small sample size is the main limitation of the current study, in particular when subgrouping the participants by task performance and the number of networks. Future replications with different task difficulty degrees in a large sample are very necessary to validate our theory and findings. We do believe, however, that this study provides an innovative method in understanding neural compensation and reserve. Also, this study is one of a few reports that explored the effect of physical fitness on neural compensatory ability. Walking speed and falling tendency have also been found associated with cognitive function in older adults in the literature (Atkinson et al., 2007; Herman et al., 2010). The relationship between the number of activated networks and walking speed provided a new mechanism for the link between physical fitness and cognitive function.

In summary, applying cognitive challenging task, identifying activated network by ICA, and combining the reserve and compensatory ability together to predict behaviors, are recommended in future studies. We believe deeper understanding of neural compensation and reserve has great potentials to prevent cognitive and brain degeneration in older adults.

## Author Contributions

LJ has participated in the whole experimental processing including fMRI task edits, subjects’ recruitment, data collection, data analysis, data summary and manuscript writing. GDP, KAH and DCS have participated in data analysis, data summary and manuscript writing. HG has provided help in data collection and manuscript revising. LW has participated in the whole experimental processing including study design, data collection, data analysis, data summary and manuscript writing. All authors have contributed in approval for the final version for publication and agreement to be accountable for the accuracy of all aspects of the work.

## Conflict of Interest Statement

The authors declare that the research was conducted in the absence of any commercial or financial relationships that could be construed as a potential conflict of interest.

## Footnotes

^1^http://www.ipaq.ki.se

^2^https://surfer.nmr.mgh.harvard.edu/fswiki/recon-all

^3^http://www.fil.ion.ucl.ac.uk/spm/software/spm8/

^4^http://mialab.mrn.org/software/gift/
